# Influence of whitebark pine decline on fall habitat use and movements of grizzly bears in the Greater Yellowstone Ecosystem

**DOI:** 10.1002/ece3.1082

**Published:** 2014-04-22

**Authors:** Cecily M Costello, Frank T van Manen, Mark A Haroldson, Michael R Ebinger, Steven L Cain, Kerry A Gunther, Daniel D Bjornlie

**Affiliations:** 1 College of Forestry and Conservation, University of MontanaMissoula, MT, 59812, USA; 2 Interagency Grizzly Bear Study Team, U.S. Geological Survey, Northern Rocky Mountain Science CenterBozeman, MT, 59715, USA; 3 Grand Teton National ParkMoose, WY, 83012, USA; 4 Bear Management Office, Yellowstone Center for ResourcesYellowstone National Park, WY, 82190, USA; 5 Large Carnivore Section, Wyoming Game & Fish DepartmentLander, WY, 82520, USA

**Keywords:** activity radius, diet, food, foraging, habitat selection, Manly–Chesson index, mast production, *Pinus albicaulis*, roads, *Ursus arctos*

## Abstract

When abundant, seeds of the high-elevation whitebark pine (WBP; *Pinus albicaulis*) are an important fall food for grizzly bears (*Ursus arctos*) in the Greater Yellowstone Ecosystem. Rates of bear mortality and bear/human conflicts have been inversely associated with WBP productivity. Recently, mountain pine beetles (*Dendroctonus ponderosae*) have killed many cone-producing WBP trees. We used fall (15 August–30 September) Global Positioning System locations from 89 bear years to investigate temporal changes in habitat use and movements during 2000–2011. We calculated Manly–Chesson (MC) indices for selectivity of WBP habitat and secure habitat (≥500 m from roads and human developments), determined dates of WBP use, and documented net daily movement distances and activity radii. To evaluate temporal trends, we used regression, model selection, and candidate model sets consisting of annual WBP production, sex, and year. One-third of sampled grizzly bears had fall ranges with little or no mapped WBP habitat. Most other bears (72%) had a MC index above 0.5, indicating selection for WBP habitats. From 2000 to 2011, mean MC index decreased and median date of WBP use shifted about 1 week later. We detected no trends in movement indices over time. Outside of national parks, there was no correlation between the MC indices for WBP habitat and secure habitat, and most bears (78%) selected for secure habitat. Nonetheless, mean MC index for secure habitat decreased over the study period during years of good WBP productivity. The wide diet breadth and foraging plasticity of grizzly bears likely allowed them to adjust to declining WBP. Bears reduced use of WBP stands without increasing movement rates, suggesting they obtained alternative fall foods within their local surroundings. However, the reduction in mortality risk historically associated with use of secure, high-elevation WBP habitat may be diminishing for bears residing in multiple-use areas.

## Introduction

Shifts in species distribution toward higher elevations are among various biological consequences of climate change (Parmesan 2006). Within communities, species can vary in their sensitivity to increasing temperatures and their rate of shift, thus climate change can cause alterations in community composition and species interactions (Van der Putten 2012). Although much work has focused on patterns of range shifts, less work has been done on the consequences of altered species interactions (Van der Putten 2012). One potential consequence is the bottom-up impact of reductions in key foods to organisms at higher trophic levels, potentially leading to decreased population viability or extinction. The loss of even a single important food can have severe impacts. For example, Pearse and Altermatt (2013) used simulations and field data to demonstrate that loss of their host plant was a significant driver for extinctions of specialist herbivores among the Lepidoptera. Similarly, LoGiudice (2006) reported that the loss of American chestnut (*Castanea dentata*) was likely a contributing factor to extirpations and reductions of Allegheny woodrat (*Neotoma magister*) populations. Although most studies of climate change impacts of food loss involve numerous food species, there are a few examples involving a single food. For example, Forcada et al. (2005) found that climate variables, closely associated with fluctuations in prey populations of krill (*Euphausia superba*), explained reductions in pup production by Antarctic fur seals (*Arctocephalus gazelle*) over a 20-year period.

**Figure 1:**
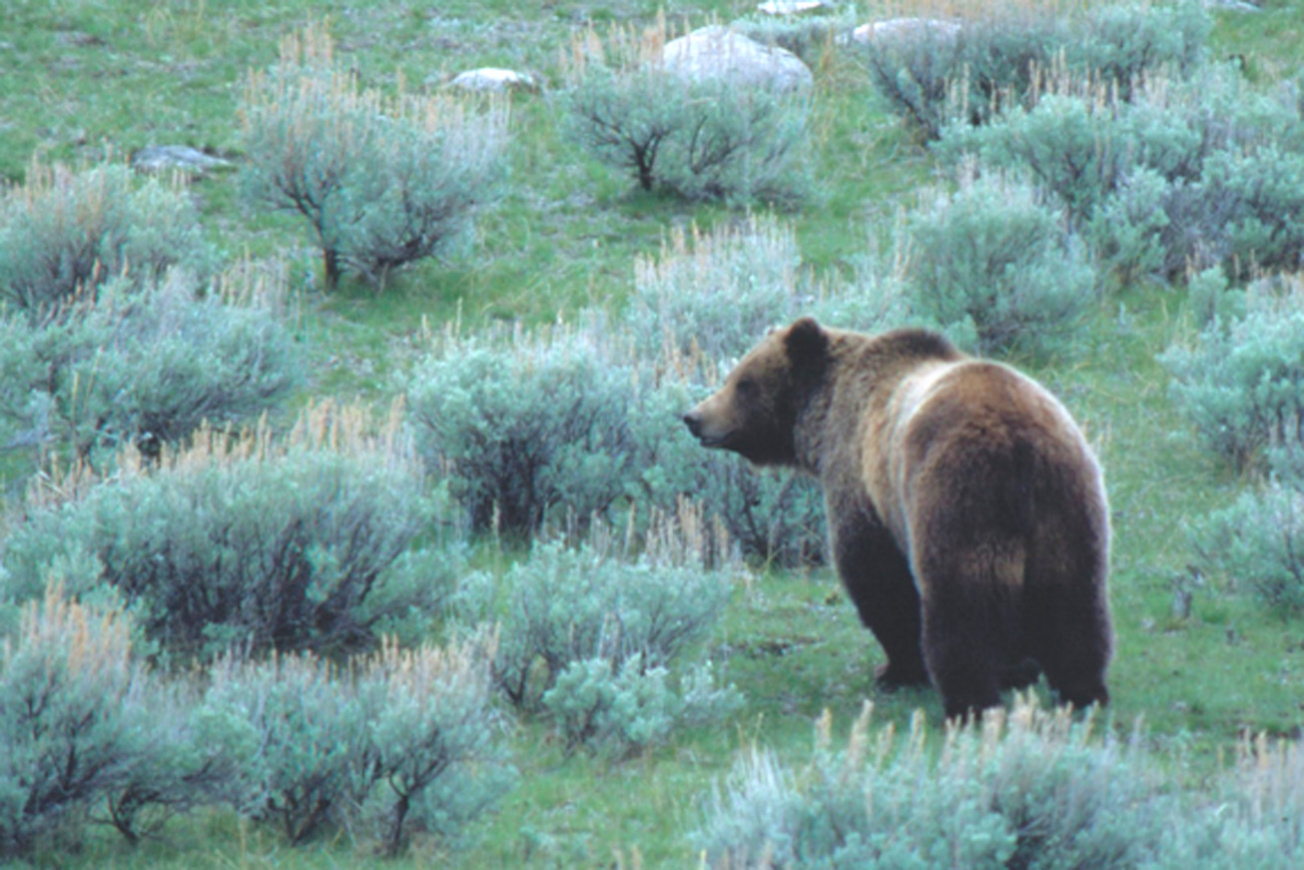
A grizzly bear (*Ursus arctos*) residing in the Greater Yellowstone Ecosystem, which encompasses portions of Wyoming, Montana, and Idaho, USA. Photo reproduced by permission of Ray Paunovich, Bozeman, Montana, USA.

Our study involves the interrelationship of two species likely affected by climate change, and the potential cascading bottom-up effect on a third species. Whitebark pine (WBP; *Pinus albicaulis*) is a long-lived conifer that occupies high-elevation sites in the northern Rocky Mountains of North America, characterized by poorly developed soils, snowy winter conditions, and highly wind-swept exposures. The range of WBP has been predicted to shrink over the next 50 years as increasing temperatures cause a shift in their lower elevational limit, above the tallest peaks in some areas (Warwell et al. 2007; Schrag et al. 2008). In contrast to this slower time frame, evidence indicates the range of mountain pine beetle (*Dendroctonus ponderosae*), a cambium-feeding insect that usually kills its pine host to reproduce, has already changed in response to climate change (Williams and Leibhold 2002; Carroll et al. 2004; Raffa et al. 2008). Historically, beetle outbreaks were infrequent in WBP forests because low winter and summer temperatures limited beetle survival and reproduction, but recent outbreaks have expanded into higher elevation WBP range (Logan and Powell 2001; Logan et al. 2010). The resulting decline in WBP could have cascading impacts, because its large, edible seeds are a food source for wildlife, including grizzly bears (*Ursus arctos*; Fig. 1). In the Greater Yellowstone Ecosystem (GYE), the most recent pine beetle eruption has caused considerable WBP mortality (Gibson et al. 2008; Macfarlane et al. 2013), resulting in a reduction in the availability of this food. Based on aerial surveys, Macfarlane et al. (2013) estimated that moderate to severe mortality had occurred within 82% of the WBP distribution in the GYE. On transects monitored annually by the Interagency Grizzly Bear Study Team (IGBST) for WBP cone production (Blanchard 1990), 73% of mature, cone-bearing sample trees died between 2002 and 2012 (Haroldson and Podruzny 2013).

Whitebark pine is a masting species (Kelly 1994) with years of good and poor seed production alternating on a 2- to 3-year cycle. Consumption by bears is correlated with this annual availability. Seeds may comprise 50–80% of fall scat volume when cone production is good, but only trace amounts when cone production is low (Kendall 1983; Mattson et al. 1991). This annual variation in WBP cone production and use has been linked with changes in grizzly bear survival rates (Haroldson et al. 2006), fecundity (Schwartz et al. 2006a,b2006b), movements (Blanchard and Knight 1991), and frequency of management actions (Mattson et al. 1992; Blanchard and Knight 1995; Gunther et al. 2004). Cone crop failures may impact nutrition, but also foraging behaviors that increase vulnerability to human-caused mortality. When WBP production is poor, grizzly bears tend to use lower elevations (Blanchard and Knight 1991; Mattson et al. 1992), where the risk of bear/human conflict is greater and survival is lower (Schwartz et al. 2010). For this grizzly bear population, which is listed as threatened under the U.S. Endangered Species Act, the potential response to the climate-related decline in WBP may have important conservation implications.

Most members of the Ursidae are opportunistic omnivores and feed on a wide variety of plant and animal foods. The foraging pattern of generalists, such as bears, often involves periodic bouts of specialization, when a particular food is used almost exclusively (Heller 1980). The relative importance of any one food depends on a variety of factors including nutritive and energetic value, abundance, distribution, predictability, and handling time (Pyke et al. 1977; McNamara and Houston 1992). On the basis of these costs and benefits, the relative importance of a food within the diet of generalists may fall within a continuum ranging from purely opportunistic use to high preference. In many American black bear (*Ursus americanus*) and Asiatic black bear (*Ursus thibetanus*) populations, for example, hard mast (e.g., acorns; *Quercus* spp.) dominates the fall diet (Hwang et al. 2002; Pelton 2003; Koike 2010). Numerous studies have shown that reproductive output is associated with annual variation in mast abundance (e.g., Costello et al. 2003; Bridges et al. 2011), and bears commonly increase movement patterns to exploit more dispersed and distant sources of mast when production is low (e.g., Garshelis and Pelton 1981; Koike et al. 2012), providing an evolutionary basis and evidence that these foods likely are on the highly preferred end of the spectrum. Although hard mast can also be an important component of grizzly bear diets (Paralikidis et al. 2009; Colangelo et al. 2012), studies linking reproductive performance and movement patterns to variable mast production are rare. This may be because grizzly bears are far more carnivorous than their black bear relatives (Schwartz et al. 2003, 2013a), allowing them to compensate for fluctuations in hard mast by consuming meat. The larger component of animal matter in grizzly bear diets is central to their ability to attain larger body sizes than black bears (Welch et al. 1997; Hilderbrand et al.^1999^; Rode et al. 2001).

Whitebark pine seeds are a food with high-fat content and moderately high digestibility (Mattson and Reinhart 1994), and they represent the only significant hard mast species consumed by bears in GYE (Gunther et al. 2014). Similar to acorns and black bears, WBP seed production has been linked with fecundity and movement rates, suggesting that WBP seeds may be a preferred fall food. However, WBP distribution is restricted to high elevations and annual abundance varies greatly. In addition, WBP cones are indehiscent; therefore, grizzlies must rely on arboreal red squirrels (*Tamiasciurus hudsonicus*) to harvest cones. Bears obtain virtually all (>90%) seeds by excavating squirrel middens (Mattson and Reinhart 1997), a manner of foraging which may require more energy than grazing or gleaning.

To understand the response of grizzly bears to the decline in this particular food, we formulated two hypotheses based on alternative explanations of the role of WBP in the fall diet. We used grizzly bear locations obtained from Global Positioning System (GPS) transmitters during 2000–2011 to investigate habitat use and movements. Our first hypothesis, predicated on the notion that WBP seeds are highly selected over other fall foods, was that grizzly bears would continue to seek this food over the study period even as availability declined. Thus, accounting for varying levels of cone production, we predicted: (1) bear selection for WBP habitats would remain stable or increase, as search time required to obtain seeds increased; (2) duration of WBP habitat use during fall would similarly remain stable or increase over time; and (3) movement rates of bears would increase over time, as the reduction in live WBP would require exploitation of more dispersed and distant stands. Additionally, we predicted that continued use of high-elevation WBP stands would also mean that bear selection of areas away from roads and human developments would also remain stable or increase.

Our second hypothesis, predicated on the notion that WBP seeds are consumed opportunistically as a part of a diverse diet determined by the relative abundance of foods, was that bears would reduce use of WBP habitat over the study period as availability of seeds declined. Again, accounting for varying cone production, we predicted: (1) bear selection for WBP habitats would decrease over time as seeds become less available; (2) duration of WBP use would decline over time, as increased competition for a declining resource would lead to diminishing returns at an earlier date; and (3) movement rates of bears would not change over time. As a consequence of reduced use of high-elevation WBP stands, we predicted that selection for areas away from roads and human developments would decrease over the study period.

## Materials and Methods

### Study area

The study area encompassed the GYE, which includes Yellowstone National Park (YNP), Grand Teton National Park, all or portions of six national forests, other federal lands, plus state, tribal, and private lands in portions of Wyoming, Montana, and Idaho. Grizzly bears in the GYE have been expanding their range and occupy approximately 50,000 km^2^ (Bjornlie et al. 2014), between 45°41′N, 111°36′W and 43°16′N, 109°21′W. Geology, hydrology, and climate of the GYE were described by Marston and Anderson (1991). Lower elevations (<1900 m) are characterized by grasslands or shrub steppes interspersed with open stands of juniper (*Juniperus scopulorum*), limber pine (*Pinus flexilis*), and Douglas-fir (*Pseudotsuga menziesii*). Douglas-fir forms the lowest elevation forest community at around 1900–2200 m. Lodgepole pine (*Pinus contorta*) dominates at mid-elevations (2400 m). Engelmann spruce (*Picea engelmannii*), subalpine fir (*Abies lasiocarpa*), and WBP form the upper tree line around 2900 m. Alpine tundra occurs at the highest reaches of all major mountain ranges (Patten 1963; Waddington and Wright 1974; Despain 1990).

### Data collection and analyses

Procedures for research trapping and collaring of grizzly bears and animal welfare protocols were previously detailed in Schwartz et al. (2006c). Since 2000, a subsample of captured bears was instrumented with GPS collars, including GEN 3 store-on-board (SOB), GEN 4 SOB, and GEN 3 Spread Spectrum Transceiver (SST) models (Telonics, Inc., Mesa, AZ, USA). Among this set, we selected bears monitored for ≥95% of the days in the peak WBP foraging season (15 August–30 September) in any year during the study period of 2000–2011 (Table 1). Although some bears continue to feed on WBP into October (Kendall 1983; Mattson, Blanchard and Knight 1991), we excluded data from this month to eliminate the confounding effects of observed predenning and denning behavior on habitat use. In addition, some transmitters were programmed to stop collecting locations in October to extend battery life, and thus, we maximized our sample size by concentrating on the peak period of WBP use.

**Table 1:** Summary of annual data from 19 whitebark pine (WBP) cone production transects (Blanchard 1990) and annual number of bears monitored with Geographic Positioning System (GPS) transmitters during fall (15 August–30 September), Greater Yellowstone Ecosystem, 2000–2011

Year	WBP cone production transects			Grizzly bears in sample		
	
	Median cone count	Rating^1^	Proportion trees alive	Total	With ≥5% WBP habitat in fall range	Outside parks, with ≥5% WBP and ≤95 secure habitat in fall range
2000	0	Poor	1.00	9	7	5
2001	13	Good	1.00	10	10	8
2002	0	Poor	1.00	3	3	3
2003	16	Good	0.92	5	5	1
2004	1	Poor	0.76	3	0	0
2005	8	Good	0.71	6	2	1
2006	22	Good	0.65	5	4	1
2007	8	Good	0.57	7	4	1
2008	1	Poor	0.43	16	7	3
2009	20	Good	0.31	14	7	3
2010	2	Poor	0.27	3	3	3
2011	12.5	Good	0.27	8	8	7
Total				89	60	36

1 WBP production was classified as poor when annual cone counts were below the overall median and good when counts were above the median (Haroldson et al. 2004).

We calculated 100% adaptive local convex hull fall home ranges (Getz et al. 2007) for each bear-year (“LoCoH.a” routine in the “adehabitatHR” package [Calenge and Fortmann-Roe 2011; ] for R [R Development Core Team 2013]). To define *a* (i.e., the distance used to define nearest neighbors, such that the sum of their distances do not exceed the value), we used 2 × the maximum distance between any two locations, except when it resulted in “orphaned holes” and failure of the routine. In those instances, we incrementally increased or decreased the multiplier of 2 to find the closest value for which the routine would succeed, from 1.0 to 2.4.

Using ArcGIS 9.3 (ESRI, Redlands, CA, USA), we calculated the proportion of WBP habitat within fall ranges using a WBP distribution map developed by Macfarlane et al. (2010). For analysis of grizzly bear selection of areas away from roads and human developments, we used secure habitat, which was defined as any area ≥4.05 ha, ≥500 m from an open or gated motorized road (Interagency Grizzly Bear Committee 1998; U.S. Fish and Wildlife Service 2007). We calculated the proportion of each fall range within secure habitat using the road layer developed by Schwartz et al. (2010).

We calculated the proportion of bear locations within WBP and secure habitats using the maps described above. Due to improved GPS technology, we observed an increasing trend in fix success over the study period. Because information from the entire study period was vital to our investigation, we needed to account for this variation in fix success in our analyses. We developed a method to assign habitat type to unsuccessful fixes and weight observations based on certainty of assignment (Appendix S1). Simulation analyses indicated our method was highly accurate and unlikely to result in erroneous inference. By utilizing successful and unsuccessful fixes, each bear was equally represented by a full, albeit weighted, set of locations. For each bear, the weights were summed and divided by the total number of fix attempts to obtain an overall weight. These overall weights were used in regression analyses.

Fourteen of 72 bears were observed during more than one year. Additionally, some data sets included multiple observations for each individual within the same year (e.g., daily movement distance). Therefore, we used likelihood ratio tests (“lrtest” routine in “lmtest” package for R; Hothorn et al. 2013) between nested fixed-effects and mixed-effects models to determine when random effects were needed to improve model fit.

For each response variable, we evaluated a set of three models using Akaike's Information Criterion adjusted for small sample size (AIC_c_; Burnham and Anderson 2002; Hurvich and Tsai 1989). Our base model accounted for potential differences by sex and annual cone production (CONES = median number of cones/tree observed on cone production transects; Blanchard 1990) and included the following variables: CONES + SEX + CONES × SEX. Evidence indicates cumulative WBP mortality increased throughout most of the study period but started waning around 2009 (Haroldson and Podruzny 2013; Mahalovich 2013; Greater Yellowstone Whitebark Pine Monitoring Working Group 2014). To assess whether bears responded to this temporal trend in WBP mortality, we compared two additional models with the base model: Base + YEAR and Base + YEAR + YEAR × CONES. The YEAR × CONES interaction in the latter model allowed us to examine whether a temporal trend, if present, differed with varying levels of cone production. We tested for potential temporal lag effects by summarizing our data in a way that allowed us to use generalized least squares models (“gls” routine in “nlme” package for R; Pinheiro et al. 2013) with an autoregression error structure (function “corAR1”). No evidence for temporal lags was found, confirming our a priori expectation that fall responses of bears depend on the current year's cone production.

Whitebark pine mortality varied across the landscape (Macfarlane et al. 2013). To account for this variability in our analyses, we used a temporally and spatially explicit index of the change in live canopy within mapped WBP habitats using the year 2000 as a baseline, derived from MODIS NDVI (normalized difference vegetation index) data (M. Ebinger, University of Montana, unpublished data). Each 250 × 250-m pixel of WBP habitat with ≥50% canopy cover received a score representing the proportional decline in NDVI compared with 2000. This index was not a direct measure of canopy mortality, but represented our best estimate of the relative impact of pine beetle kill (and other factors, such as wildfire) on WBP habitat. Accordingly, we adjusted the mapped proportion of WBP habitat within each fall home range by multiplying it by (1 − ΔNDVI). Thus, a greater decline in NDVI resulted in greater downward adjustment of proportion of WBP habitat within each range.

### Habitat selection

For analyses of habitat selection, we used the Manly–Chesson standardized index of selectivity (MC index; Manly et al. 1972; Chesson 1978) as our response variable. This index quantified relative use of each habitat by individual bears relative to its availability within the fall range, according to:


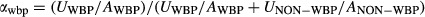


where *U*_WBP_ is the proportion of locations within WBP habitat, *U*_NON-WBP_ is the proportion of locations not in WBP habitat, *A*_WBP_ is the proportion of WBP habitat in the fall range, and *A*_NON-WBP_ is the proportion of the fall range not in WBP habitat. This index varies from 0 to 1, corresponding with exclusive selection against and for WBP habitat, respectively. With only two habitat types, an index of 0.5 would indicate use equal to availability (i.e., no selection). We calculated a MC index for WBP habitat, using the proportion of WBP habitat within the range. We also calculated an MC index for impact-adjusted WBP habitat using the revised proportion adjusted by ΔNDVI. Finally, we calculated an independent MC index for secure habitat, because WBP and secure habitats overlapped.

We used multiple linear regression (“glm” in R) with MC index as the response. Although MC indices were bounded by 0 and 1, mean values were centered, allowing us to use conventional linear regression (family = “gaussian”) without producing predicted values or confidence intervals outside of these bounds. For the analyses of WBP and impact-adjusted WBP habitats, we excluded bears for which proportion of WBP habitat was <0.05. Similarly, for the analysis of secure habitat, we excluded bears for which proportion of secure habitat was >0.95. First, for most of these bears, MC indices were not calculable due to zeros present in the denominator (i.e., there was zero availability for one of the two possible habitats). Secondly, MC indices can be erratic when the proportions of habitat and use are both extreme nonzero values. To illustrate, a simulated difference of only 0.01 in the proportion of bear locations within WBP habitat changed the resulting MC index by as much as 0.50 when habitat proportion was <0.05, whereas a simulated 0.01 difference changed the index by ≤0.08 when habitat proportion was ≥0.05. For the analysis of secure habitat selection, we restricted our sample to bears residing partially or entirely outside of national parks (i.e., those bears more vulnerable to human-caused mortality).

### Timing and duration of WBP use

For the analysis of timing of use of WBP habitat, we defined days of WBP use as those days when ≥14% of locations were within WBP habitat, corresponding to the minimum detectable level of use within a day for bears with the longest fix interval (1 of 7 daily locations). We used quantile regression (“quantreg” package in R; Koenker 2011) with day-of-year as the response. Use of quantile regression also allowed us predict median date of use, as well as the 10th and 90th percentiles to examine potential changes in early and late dates of WBP use, respectively.

### Daily and seasonal movement patterns

For the analyses of movements, we first estimated fall and daily location centers (i.e., median x and y coordinates) for each bear, which allowed us to equalize the number of observations among individuals regardless of fix interval. These daily estimates were used to calculate two indices of movement. First, we calculated the net distance moved between two successive days as a measure of the rate of travel. Second, we calculated the distance between the fall range center and each daily center (i.e., activity radius; Dice and Clark 1953) as a measure of the area roamed by bears. We used mixed-effects multiple linear regression (“lme” routine in “nlme” package for R; Pinheiro et al. 2013) for these analyses. Response variables were log-transformed to fit a normal distribution, thus estimates were based on the median.

## Results

Our analyses were based on 52,321 successful GPS locations of 72 individuals during 89 bear-years (hereafter bears). Weights applied to bears to account for fix success were 0.87–1.0 for use of WBP habitats and 0.91–1.0 for the use of secure habitat. Fall home-range size ranged from 26 to 354 km^2^ for females (*n* = 42), and 58 to 1381 km^2^ for males (*n* = 47). Fall ranges of sampled bears were well distributed across the study area throughout the study period.

### WBP habitat selection

**Figure 2:**
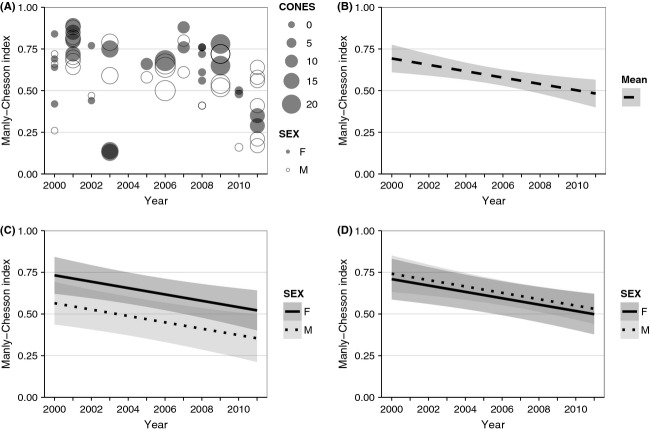
Manly–Chesson indices of grizzly bear selection of whitebark pine (WBP) habitat, Greater Yellowstone Ecosystem, 2000–2011: (A) observed values, by sex and annual median WBP cone count (CONES); (B) model-predicted estimates (±95% confidence interval [CI]) when sex and CONES were kept constant at their means; (C) model-predicted estimates (±95% CI) by sex averaging for poor WBP production (CONES = 1); and (D) model-predicted estimates (±95% CI) by sex averaging for good WBP production (CONES = 16).

Fall ranges of 21 bears did not encompass any mapped WBP habitat, and ranges of another 8 bears encompassed <5% WBP habitat. Together, these 29 bears represented 33% of the sample. For the remaining 60 bears, values were 0.05–0.71 for the proportion of WBP habitat within the fall range, 0.01–0.89 for the proportion of locations within WBP habitat, and 0.13–0.89 for the MC index for WBP (Fig. 2A). There was no detectable trend in proportion of WBP habitat in fall ranges over the study period (*r* = −0.21, *P* = 0.11), nor was there a correlation between MC indices and proportion of WBP habitat in fall ranges (*r* = 0.01, *P* = 0.94). Most bears (72%) had a MC index >0.50, indicating selection for WBP habitats.

The model Base + YEAR ranked highest in AIC_c_ (Table 2). Coefficients for SEX (*β* = −0.181, SE = 0.081), YEAR (*β* = −0.019, SE = 0.006) and CONES × SEX (*β* = 0.013, SE = 0.007) had confidence intervals (CI) that did not overlap zero (Appendix S2). Mean MC index was negatively associated with YEAR. Keeping other variables constant at their means, model-predicted mean MC index declined from 0.69 to 0.48 over the 12-year study period, a difference of 0.21 (95% CI: 0.04–0.38; Fig. 2B). Overall, mean MC index was positively associated with CONES; however, this effect was only apparent among males (Fig. 2C,D). Keeping year constant at the mean, model-predicted MC index was 0.64 for males in an average year of good production (CONES = 16) and 0.46 in an average year of poor production (CONES = 1), a difference of 0.18 (95% CI: −0.01–0.38). Among females, model-predicted MC index was 0.60 in a year of good production and 0.63 in a year of poor production, with the confidence interval of the difference containing zero (95% CI: −0.22–0.17).

**Table 2:** Akaike information criterion (AIC_c_) model selection results for regression models predicting responses of grizzly year to the decline of whitebark pine (WBP), Greater Yellowstone Ecosystem, 2000–2011

Response	Regression model type	Model	K	AIC_c_	ΔAIC_c_	AIC_c_ weight
Manly–Chesson index for WBP habitat	Linear	Base^1^ + YEAR	6	−24.33	0	0.73
		Base + YEAR + YEAR × CONES^2^	7	−22.14	2.19	0.25
		Base	5	−17.39	6.95	0.02
Manly–Chesson index for impact-adjusted WBP habitat	Linear	Base + YEAR	6	−24.92	0	0.70
		Base + YEAR + YEAR × CONES	7	−22.73	2.19	0.24
		Base	5	−20.03	4.89	0.06
Day-of-year (median)	Quantile	Base + YEAR + YEAR × CONES	6	14,703.62	0	0.87
		Base + YEAR	5	14,707.46	3.84	0.13
		Base	4	14,736.72	33.09	0
Day-of-year (10th percentile)	Quantile	Base + YEAR	6	14,938.70	0	0.70
		Base + YEAR + YEAR × CONES	5	14,940.35	1.65	0.30
		Base	4	14,961.16	22.46	0
Day-of-year (90th percentile)	Quantile	Base + YEAR	5	14,724.12	0	0.61
		Base + YEAR + YEAR × CONES	6	14,725.59	1.47	0.29
		Base	4	14,727.75	3.63	0.01
Log (distance)	Mixed-effects linear	Base	6	10,678.67	0	0.56
		Base + YEAR	7	10,679.81	1.14	0.32
		Base + YEAR + YEAR × CONES	8	10,681.82	3.15	0.12
Log (radius)	Mixed-effects linear	Base	6	7469.04	0	0.65
		Base + YEAR	7	7471.01	1.97	0.24
		Base + YEAR + YEAR × CONES	8	7472.68	3.64	0.11
Manly–Chesson index for secure habitat	Linear	Base + YEAR + YEAR × CONES	7	−22.37	0	0.57
		Base + YEAR	6	−21.09	1.27	0.30
		Base	5	−19.45	2.92	0.13

1 Base model = SEX + CONES + SEX × CONES.

2 CONES = median number of cones/tree observed on cone production transects.

**Figure 3:**
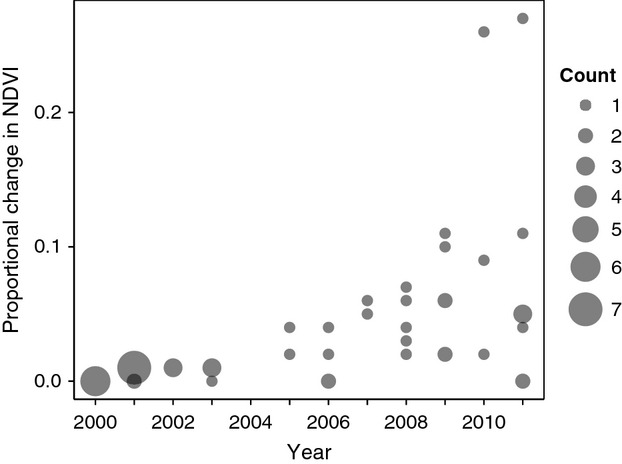
Impact of WBP decline within individual bear ranges by year, estimated as proportional negative change in MODIS normalized difference vegetation index (NDVI), using 2000 as a baseline.

As expected, indices of WBP impact (mean ΔNDVI within bear ranges) were positively associated with year (*r* = 0.52, *P* < 0.001, *n* = 60; Fig. 3). Most indices were between 0 and 0.11; however, two outliers of 0.26 and 0.27 were observed during 2010 and 2011. Unlike all other fall home ranges that displayed gradual changes in NDVI over time, these two outliers displayed a rapid and widespread change, likely indicating the impact was caused by a wildfire in 2007. Among individuals, mean ΔNDVI scores of bear locations ranged from 0 to 0.30 and were highly correlated with mean ΔNDVI scores within their range (*r* = 0.92, *P* < 0.001, *n* = 60). A paired *t*-test revealed no differences between mean ΔNDVI of WBP habitat available in fall ranges versus mean ΔNDVI of WBP habitat associated with bear locations (difference = −0.002, SE = 0.003, *t* = −0.72, df = 59, *P* = 0.47).

Estimated proportion of impact-adjusted WBP habitat within ranges, as compared with total WBP habitat, declined by 0–0.10. Resulting MC indices increased by 0–0.10. Based on these revised MC indices, the model Base + YEAR still ranked highest according to AIC_c_ (Table 2) and the same coefficients had CIs different from zero. Keeping other variables constant at their means, model-predicted mean MC index declined from 0.69 to 0.51 over the 12-year study period, a difference of 0.18 (95% CI: 0.02–0.35). Relationships of sex and WBP production with MC index were similar to those predicted from the model based on unadjusted WBP habitat.

### Timing of use of WBP habitat

Use of WBP habitats was observed throughout the fall period. Summed across years, 43–75% of the 60 bears used WBP habitat on any given day-of-year for a total of 1779 bear-days of use. Total use per bear ranged from 1 day to the full 47 days, with a mean of 30 days. Mean consecutive days of use per bear was only 12 days, indicating that many bears left and returned to WBP habitat over the season.

The model Base + YEAR + CONES × YEAR ranked as the single top model for predicting the median date of WBP use and accounted for 87% of AIC_c_ model weight (Table 2). Coefficients for YEAR (*β* = 0.778, SE = 0.201) and CONES × SEX (*β* = 0.300, SE = 0.139) were the only coefficients with CIs that did not overlap zero (Appendix S2). Predicted median date of WBP use changed over the study period, but this change was primarily observed when cone production was poor. Averaging for SEX and estimating for poor WBP cone production (CONES = 1), predicted median date was 8.2 days later (95% CI: 2.9–13.5) comparing 2000 to 2011, shifting from about 7 September to 15 September. Estimating for good WBP cone production (CONES = 16), there was no detectable change in median date over the study period (difference = 3.3 days, 95% CI: −1.4–8.0); predicted median date was 10 September. Comparing within YEAR, there were no detectable differences between males and females, or between good and poor years of WBP cone production.

The model Base + YEAR was the top-ranked model for estimating the 10th percentile of date of use (early use; Table 2). The CI for the coefficient YEAR (*β* = 0.330, SE = 0.104) did not overlap zero. Averaging for SEX and CONES, predicted early date of WBP use was 4.2 days later (95% CI: 2.0–4.7) comparing 2001 to 2011, shifting from about 20 August to 24 August. Comparing within YEAR, there were no detectable differences between males and females, or between good and poor years of WBP cone production.

Both models with YEAR ranked above the base model for predicting the 90th percentile of date of WBP use (late use), with a combined AIC_c_ weight of 0.90 (Table 2). However, coefficients in all models had CIs that overlapped zero. There were no detectable differences in late date of WBP use relative to the predictors. Mean predicted date of late use was 27 September.

### Movement patterns

Among the 60 bears, daily movement distances were highly variable, ranging from 0 to 25.9 km (*n* = 2757). The top-ranked model was the base model, with 98% of AIC_c_ model weight (Table 2). The CIs for all coefficients overlapped zero (Appendix S2). Based on this model, median daily movement distance was 1.4 km (95% CI: 1.2–1.6). Daily activity radii were also highly variable and ranged from 0.1 to 67.5 km (*n* = 2818). The base model had most support (AIC_c_ weight = 0.65; Table 2); however, the CIs for all coefficents in all models overlapped zero. Based on the top model, median activity radius was 3.9 km (95% CI: 3.4–4.5).

### Selection of secure habitat

**Figure 4:**
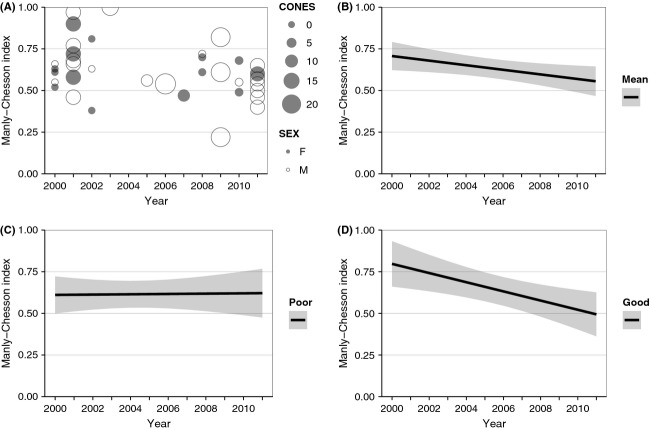
Manly–Chesson indices of grizzly bear selection of secure habitat for bears residing outside of nationals parks, Greater Yellowstone Ecosystem, 2000–2011: (A) observed values, by sex and annual median WBP cone count (CONES); (B) model-predicted estimates (±95% confidence interval [CI]) when sex and CONES were kept constant at their means; (C) model-predicted estimates (±95% CI) averaging for sex and poor WBP production (CONES = 1); and (D) model-predicted estimates (±95% CI) averaging for sex and good WBP production (CONES = 16).

**Figure 5:**
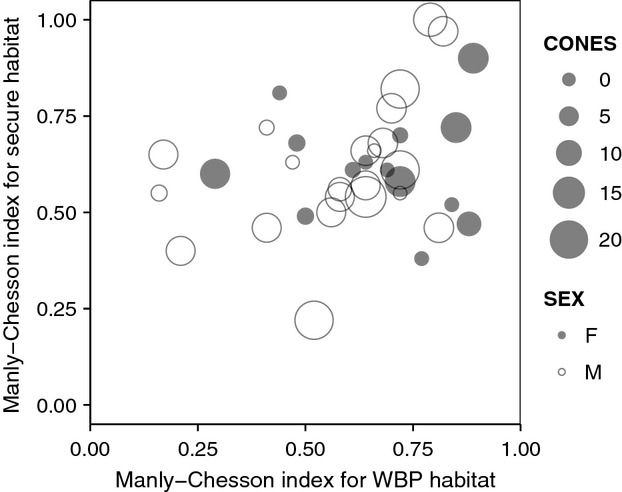
Relationship between Manly–Chesson index for selection of whitebark pine (WBP) habitat and Manly–Chesson index for selection of secure habitat, Greater Yellowstone Ecosystem, 2000–2011. Values are displayed according to sex and annual median WBP cone count (CONES).

Among the 60 bears with ≥5% WBP habitat within their fall ranges, 52 resided outside of the national parks. Among these, ranges of 10 bears were entirely comprised of secure habitat and ranges of another six bears encompassed >95% secure habitat. For the remaining 36 bears, values were 0.29–0.94 for the proportion of fall ranges within secure habitat, 0.32–1.00 for the proportion of locations, and 0.22–1.00 for MC index of selectivity (Fig. 4A). There was a positive trend in the proportion of fall ranges in secure habitat over the study period (*r* = 0.35, *P* = 0.04, *n* = 36). The MC index was not correlated with this proportion (*r* = 0.20, *P* = 0.24, *n* = 36). Most bears (78%) had an MC index of >0.50, indicating selection for secure habitat. MC index for secure habitat was not correlated with MC index for WBP use (*r* = 0.27, *P* = 0.12, *n* = 36; Fig. 5).

The model Base + YEAR + CONES × YEAR ranked highest in the model set with an AIC_c_ weight of 0.57 (Table 2). Among the model-averaged coefficients, only CONES × YEAR (*β* = −0.002, SE = 0.001) had a CI narrowly different from zero (Appendix S2). Based on this model, we did not detect a change in predicted MC index through the study period, when averaging for SEX and CONES (difference = 0.15, 95% CI: −0.02–0.32; Fig. 4B). When averaging for SEX and poor WBP production (CONES = 1), mean predicted MC index remained stable at 0.61 throughout the study period (Fig. 4C). When averaging for SEX and good WBP production (CONES = 16), predicted MC index declined from 0.80 to 0.49, a difference of 0.31 (95% CI: 0.03–0.57; Fig 4D).

## Discussion

The behavioral response of grizzly bears to reduced WBP seed availability is an important component of our understanding of the potential impacts of WBP decline on the GYE grizzly population. We documented this response by testing two alternative hypotheses. The first, predicated on the concept that WBP seeds are highly selected over other fall foods, was that grizzly bears would continue to seek this food even as availability declined. Our second hypothesis, predicated on the concept that WBP seeds are consumed opportunistically as a part of a diverse diet, was that bears would reduce use of WBP habitat as availability of seeds declined. Results supported our second hypothesis. A negative trend in selection of WBP habitats was evident over the study period, even in analyses involving impact-adjusted WBP habitat. When WBP production was poor, dates of early and peak use of WBP habitat by grizzlies shifted 5–8 days later over the study period, thus shortening the period of use. Reduced use of WBP following disturbance is not unprecedented in the GYE. Podruzny et al. (1999) reported that bear feeding activity within WBP habitats on Mount Washburn decreased disproportionately more than squirrel midden abundance following WBP loss from the large-scale 1988 wildfires. The authors attributed this disparity to a decline in midden size, noting that excavation of smaller middens may offer less energetic profit. Given this evidence that the value of WBP seeds to bears is conditional not only on their harvest by red squirrels, but also on the profitability of excavating middens, it is not surprising that our results did not support the hypothesis that WBP seeds are highly selected over other fall foods by grizzly bears.

Although selection of WBP habitats declined, bears did not abandon the resource. Individual MC indices above 0.5 were observed during all years and the mean predicted MC index did not fall much below this level for females, or for males during good WBP years. In addition, about one third of bears were observed to use stands where impact exceeded 10%. However, at least two bears were possibly drawn to these stands by the postfire availability of early successional food plants. In 2011, Podruzny (2012) also documented continued use of WBP stands by two female grizzlies intensively ground-monitored northwest of YNP, despite approximately 50% beetle-caused mortality. WBP accounted for 16% of observed feeding activity and 27% of dry digestible matter in collected scats (Podruzny 2012).

Unlike previous bear studies documenting increased movement rates associated with shortage of hard mast (Garshelis and Pelton 1981; Blanchard and Knight 1991; Koike et al. 2012), we detected no temporal trend in movement indices over the study period. Because bears did not roam over larger areas or canvass more within their fall range, these data suggest they foraged on alternative foods within their fall ranges. This finding also supports our second hypothesis and is consistent with previous studies that have demonstrated a wide breadth of diet items for Yellowstone grizzly bears and considerable dietary plasticity (Mealey 1980; Mattson, Blanchard and Knight 1991; Schwartz et al. 2013a). In a recent, comprehensive review of foods consumed by grizzly bears in the GYE, at least 266 species were identified, including 175 plant and 83 animal foods (Gunther et al. 2014).

Seeds of WBP have long been considered a fall staple for grizzly bears in the GYE. Widespread use of WBP (Kendall 1983; Mattson, Blanchard and Knight 1991), along with evidence for various population-level impacts of poor WBP production (Haroldson et al. 2006; Schwartz et al. 2006a,b2006b), may have lead to the implicit assumption that most, if not all, grizzly bears consumed WBP seeds as part of their fall diet, at least in years of high productivity. Thus, our results showing that one third of the grizzly bears in our sample made little or no use of WBP habitat, many during good WBP years, were unexpected. These observations occurred throughout the study period and were equally divided between males and females, and between good and poor years of WBP production. Unlike previous studies, the use of GPS collars allowed us to document use (or nonuse) of WBP habitats at a fine temporal scale. Although it is likely that not all WBP stands have been accurately mapped, it is doubtful that mapping error alone would account for the lack of WBP habitat in the various fall ranges we documented. Our results suggest a considerable number of bears feed almost exclusively on other foods during fall, even during years of good WBP production.

The presence of intrapopulation variation in feeding strategies (i.e., diet specialization) is likely within an area as immense as the GYE. Using stable isotope analysis, Edwards et al. (2011) identified three foraging groups within a grizzly population in the Canadian arctic, ranging from near-complete herbivory to near-complete carnivory. Similar diet specialization was proposed by Mealey (1980), who identified three foraging economies within YNP: valley/plateau, mountain, and lake. The valley/plateau economy centered on grassland habitats surrounded by lodgepole pine forests and involved substantial consumption of meat derived primarily from ungulates and rodents, as well as roots and corms. The locations of our observed WBP-deficient fall ranges (inside, west, and south of YNP) correspond well with the locales described by Mealey (1980), namely Hayden, Pelican, and Lamar valleys, along with Cougar Creek Flat. These areas have high ungulate densities, therefore meat is likely the primary food resource for resident bears during fall. From field surveys around Yellowstone Lake during 2007–2009, Fortin et al. (2013) documented that ungulate remains represented 20–50% of the grizzly diets during August and September, often acquired by usurping wolf (*Canis lupus*) kills. Recent analyses also indicated greater levels of animal consumption in autumns with poor WBP production (Schwartz et al. 2013a). In addition, bears can be attracted to areas outside of the national parks during fall, when ‘gut piles’, wounding loss, and other remains left by elk hunters become available, regardless of WBP production (Haroldson et al. 2004). Whether obtained from predation or scavenging wolf- or hunter-killed carcasses, meat is clearly an important source of nutrition for grizzlies in the GYE. Together, three stable isotope analyses have indicated that animal matter accounts for roughly half of annual male diets and 40% of annual female diets (Jacoby et al. 1999; Felicetti et al. 2003; Fortin et al. 2013).

Greater carnivory among males compared with females might explain reduced male selection of WBP habitats during years of poorer WBP production. When WBP seeds are less abundant, the strategy of males may be to concentrate on meat resources because their larger body size allows them to dominate at carcasses, whereas the strategy of females may be to reduce direct competition with larger males by focusing more time on WBP foraging. Blanchard and Knight (1991) postulated that adult males might displace other cohorts from productive WBP stands during years of poor production, but our results suggest that intersexual competition within WBP habitats may be higher during years of good WBP production.

As predicted for our second hypothesis, along with reduced use of WBP habitat over time, we also detected a corresponding negative trend in selectivity of secure habitat. However, this trend was only apparent when WBP cone production was good (specifically when CONES ≥ 11). During the early years of the study period, selectivity of secure habitat was greater during years of good cone production compared to poor. This is consistent with previous studies that indicated WBP foraging in higher elevations, in years of high cone availability, made bears less vulnerable to human-caused mortality (Blanchard and Knight 1991; Mattson et al. 1992). By the end of the study period, there was little difference in selectivity of secure habitats between good and poor years, perhaps indicating the benefit of this effect was diminished. Nonetheless, the lack of a relationship between the selection of WBP habitats and the selection of secure habitats suggests that bears were not necessarily compelled to use less secure habitats as a direct response to WBP decline. On average, 48% of fall ranges were comprised of secure habitat outside of WBP forests, indicating most bears had ample opportunities to use secure habitats, even in the absence of WBP foraging. Consequently, most bears selected for secure habitat, irrespective of the intensity of WBP use. Among our sample of bears with WBP habitat within their fall range, 13% used ranges entirely within national parks, 27% used ranges that encompassed ≥95% secure habitat, and 47% selected for secure habitat when nonsecure habitat was present in their range. In other words, only the remaining 13% selected for nonsecure habitat. These results strengthen the supposition put forth by Schwartz et al. (2010) in their analysis of hazards to Yellowstone grizzly bear survival. Although these authors found that bears shifted to lower elevations during years of poor WBP production, they concluded that this elevation shift did not itself predispose bears to increased mortality. Instead, they found that bears shifting to lower elevations that had been altered by humans were exposed to more risk, whereas those bears shifting to lower elevations in secure habitat were not subject to increased risk.

Finally, previous studies have shown that transient brown bears, such as dispersing subadult males, often experience greater mortality rates because of increased encounters with anthropogenic features, such as roads and developments (Kaczensky et al. 2003; Krofel et al. 2012). Similarly, increased human-caused mortality of black bears has also been correlated with low hard mast abundance, presumably at least partially attributable to greater mobility (Noyce and Garshelis 1997; Ryan et al. 2007). We did not detect any changes in grizzly bear mobility associated with the period of WBP decline. Therefore, it is unlikely that WBP decline intensified the potential for human-caused mortality associated with greater movement rates.

Theory (Clavel et al. 2011) and field studies (Warren et al. 2001; Menéndez et al. 2006; Devictor et al. 2008) suggest habitat generalists typically fare better than habitat specialists in response to disturbance, including climate change. Under various influences of a changing climate, the long-term outlook for WBP – a habitat specialist – is uncertain; however, range contraction is generally predicted (Warwell et al. 2007; Schrag et al. 2008; Logan et al. 2010). The recent irruption of mountain pine beetles, likely stimulated by warming temperatures, has already caused considerable mortality in WBP populations in the GYE. Our study provides some indirect evidence that the cascading effect of this decline on grizzly bears—a habitat generalist—was not as severe. Diet plasticity is central to the evolutionary strategy of grizzly bears and allows them to occupy the largest and most diverse range of any bear species (Schwartz et al. 2003, 2013b; Van Daele et al. 2012). This plasticity is evident in the temporal and spatial variability of WBP habitat use we observed in the GYE. Notably, many grizzly bears in our sample did not make any use of WBP seeds, even prior to the decline. Over the period of WBP decline, the remaining bears reduced use of WBP stands without increasing movement rates, suggesting they obtained alternative foods within their local surroundings. However, the reduction in risk of mortality and conflicts historically associated with use of secure, high-elevation WBP habitat, during years of good WBP productivity, may be diminishing among the subpopulation of bears residing in multiple-use areas.

Data from WBP cone production transects (Haroldson and Podruzny 2013), aerial surveys (Mahalovich 2013), and field surveys (Greater Yellowstone Whitebark Pine Monitoring Working Group 2014) indicate a waning of the pine beetle epidemic in the GYE. Therefore, the timing of our study likely encapsulated years before, during, and after the peak of this particular epidemic. Despite the declining trend, grizzly bears continued to select for WBP habitats during our study period, so continued monitoring would provide valuable insight into potential impacts of any additional loss of WBP resources.

## Supporting Information

**Appendix S1.** Assigning habitat to unsuccessful GPS fixes: rationale, methodology, and evaluation of bias and accuracy.

**Appendix S2.** Coefficients for top-ranked models, based on Akaike Information Criterion (AIC_c_), predicting various grizzly bear responses, as a function of annual estimates of WBP cone production, sex and year, Greater Yellowstone Ecosystem, 2000–2011

## References

[b1] Bjornlie DD, Thompson DJ, Haroldson MA, Schwartz CC, Gunther KA, Cain SL (2014). Methods to estimate distribution and range extent of grizzly bears in the Greater Yellowstone Ecosystem. Wildl. Soc. Bull.

[b2] Blanchard B, Schmidt WC, McDonald KJ (1990). Relationship between whitebark pine cone production and fall grizzly bear movements. Proceedings of symposium on whitebark pine ecosystems: ecology and management of a high mountain resource.

[b3] Blanchard BM, Knight RR (1991). Movements of Yellowstone grizzly bears. Biol. Conserv.

[b4] Blanchard BM, Knight RR (1995). Biological consequences of relocating grizzly bears in the Yellowstone Ecosystem. J. Wildl. Manag.

[b5] Bridges AS, Vaughan MR, Fox JA (2011). Reproductive ecology of American black bears in the Alleghany Mountains of Virginia, USA. J. Wildl. Manag.

[b6] Burnham KP, Anderson DR (2002). Model selection and multimodel inference: a practical information-theoretic approach.

[b7] Calenge C, Fortmann-Roe S (2011). http://cran.r-project.org/web/packages/adehabitatHR.

[b8] Carroll AL, Taylor SW, Régnière J, Safranyik L, L Shore T, Brooks JE, Stone JE (2004). Effects of climate change on range expansion by the mountain pine beetle in British Columbia. Mountain pine beetle symposium: challenges and solutions.

[b9] Chesson J (1978). Measuring preference in selective predation. Ecology.

[b10] Clavel J, Julliard R, Devictor V (2011). Worldwide decline of specialist species: toward a global functional homogenization?. Front. Ecol. Evol.

[b11] Colangelo P, Loy A, Huber D, Gomercic T, Taglianti AV, Ciucci P (2012). Cranial distinctiveness in the Apennine brown bear: genetic drift effect or ecophenotypic adaptation?. Biol. J. Linn. Soc.

[b12] Costello CM, Jones DE, Inman RM, Inman KH, Thompson BC, Quigley HB (2003). Relationship of variable mast production to American black bear reproductive parameters in New Mexico. Ursus.

[b13] Despain DG (1990). Yellowstone vegetation: consequences of environment and history in a natural setting.

[b14] Devictor V, Julliard R, Jiguet F (2008). Distribution of specialist and generalist species along spatial gradients of habitat disturbance and fragmentation. Oikos.

[b15] Dice LR, Clark PJ (1953). The statistical concept of home range as applied to the recapture radius of the deermouse (Peromyscus), Laboratory of Vertebrate Biology Contribution Number 62.

[b16] Edwards MA, Derocher AE, Hobson KA, Branigan M, Nagy JA (2011). Fast carnivores and slow herbivores: differential foraging strategies among grizzly bears in the Canadian Arctic. Oecologia.

[b17] Felicetti LA, Schwartz CC, Rye RO, Haroldson MA, Gunther KA, Robbins CT (2003). Use of sulfur and nitrogen stable isotopes to determine the importance of whitebark pine nuts to Yellowstone grizzly bears. Can. J. Zool.

[b18] Forcada J, Trathan PN, Reid K, Murphy EJ (2005). The effects of global climate variability in pup production of Antarctic fur seals. Ecology.

[b19] Fortin JK, Schwartz CC, Gunther KA, Teisberg JE, Haroldson MA, Evans MC (2013). Dietary adjustment of grizzly bears and American black bears in Yellowstone National Park. J. Wildl. Manag.

[b20] Garshelis DL, Pelton MR (1981). Movements of black bears in the Great Smoky Mountains National Park. J. Wildl. Manag.

[b21] Getz WM, Fortmann-Roe S, Cross PC, Lyons AJ, Ryan SJ, Wilmers CC (2007). LoCoH: nonparameteric kernel methods for constructing home ranges and utilization distributions. PLoS ONE.

[b22] Gibson K, Skov K, Kegley S, Jorgensen C, Smith S, Witcosky J (2008). Mountain pine beetle impacts in high elevation five-needle pines: current trends and challenges.

[b23] Greater Yellowstone Whitebark Pine Monitoring Working Group (2014). Summary of preliminary step-trend analysis from the Interagency Whitebark Pine Long-term Monitoring Program—2004-2013: Prepared for the Interagency Grizzly Bear Study Team, Natural Resource Data Series NPS/GRYN/NRDS–2014/600.

[b24] Gunther KA, Haroldson MA, Cain SL, Copeland J, Frey K, Schwartz CC (2004). Grizzly bear-human conflicts in the Yellowstone Ecosystem. Ursus.

[b25] Gunther KA, Shoemaker R, Frey K, Haroldson MA, Cain SL, van Manen FT (2014). Dietary breadth of grizzly bears in the Greater Yellowstone Ecosystem. Ursus.

[b26] Haroldson MA, Podruzny S, van Manen FT, Haroldson MA, West K (2013). Whitebark pine production. Yellowstone grizzly bear investigations: annual report of the Interagency Grizzly Bear Study Team, 2012.

[b27] Haroldson MA, Schwartz CC, Cherry S, Moody DS (2004). Possible effects of elk harvest on fall distribution of grizzly bears in the Greater Yellowstone Ecosystem. J. Wildl. Manag.

[b28] Haroldson MA, Schwartz CC, White GC (2006). Survival of independent grizzly bears in the Greater Yellowstone Ecosystem, 1983–2001. Wildl. Monogr.

[b29] Heller R (1980). On optimal diet in a patchy environment. Theor. Popul. Biol.

[b30] Hilderbrand GV, Schwartz CC, Robbins CT, Jacoby ME, Hanley TA, Authur SM (1999). The importance of meat, particularly salmon, to body size, population productivity, and conservation of North American brown bears. Can. J. Zool.

[b31] Hothorn T, Zeileis A, Farebrother RW, Cummins C, Millo G, Mitchell D (2013). http://cran.r-project.org/web/packages/lmtest/.

[b32] Hurvich CM, Tsai C-L (1989). Regression and time series model selection in small samples. Biometrika.

[b33] Hwang M, Garshelis DL, Wang Y (2002). Diets of Asiatic black bears in Taiwan, with methodological and geographical comparisons. Ursus.

[b34] Interagency Grizzly Bear Committee (1998). Interagency Grizzly Bear Committee task force report: grizzly bear/motorized access management, revised 1998.

[b35] Jacoby ME, Hilderbrand GV, Servheen C, Schwartz CC, Arthur SM, Hanley TA (1999). Trophic relations of brown and black bears in several western North American ecosystems. J. Wildl. Manag.

[b36] Kaczensky P, Knauer F, Krze B, Jonozovic M, Adamic M, Gossow H (2003). The impact of high speed, high volume traffic axes on brown bears in Slovenia. Biol. Conserv.

[b37] Kelly D (1994). The evolutionary ecology of mast seeding. Trends Ecol. Evol.

[b38] Kendall KC (1983). Use of pine nuts by grizzly and black bears in the Yellowstone area. Int. Conf. Bear Res. Manage.

[b39] Koenker R (2011). http://cran.r-project.org/web/packages/quantreg/.

[b40] Koike S (2010). Long-term trends in food habits of Asiatic black bears in the Misaka Mountains on the Pacific coast of central Japan. Mamm. Biol.

[b41] Koike S, Kozakai C, Nemoto Y, Masaki T, Yamazaki K, Abe S (2012). Effect of hard mast production on foraging and sex-specific behavior of the Asiatic black bear (*Ursus thibetanus*. Mamm. Stud.

[b42] Krofel M, Jonozovič M, Jerina K (2012). Demography and mortality patterns of removed brown bears in a heavily exploited population. Ursus.

[b43] Logan JA, Powell JA (2001). Ghost forests, global warming, and the mountain pine beetle. Am. Entomol.

[b44] Logan JA, Macfarlane WW, Willcox L (2010). Whitebark pine vulnerability to climate-driven mountain pine beetle disturbance in the Greater Yellowstone Ecosystem. Ecol. Appl.

[b45] LoGiudice K (2006). Toward a synthetic view of extinction: a history lesson from a North American rodent. Bioscience.

[b46] Macfarlane WW, Logan JA, Kern WR (2010). Using the landscape assessment system (LAS) to assess mountain pine beetle-caused mortality of whitebark pine, Greater Yellowstone Ecosystem, 2009: project report.

[b47] Macfarlane WW, Logan JA, Kern WR (2013). An innovative aerial assessment of Greater Yellowstone Ecosystem mountain pine beetle-caused whitebark pine mortality. Ecol. Appl.

[b48] Mahalovich MF (2013). Grizzly bears and whitebark pine in the Greater Yellowstone Ecosystem. Future status of whitebark pine: blister rust resistance, mountain pine beetle, and climate change.

[b49] Manly BJF, Miller P, Cook L (1972). Analysis of a selective predation experiment. Am. Nat.

[b50] Marston RA, Anderson JE (1991). Watersheds and vegetation of the Greater Yellowstone Ecosystem. Conserv. Biol.

[b51] Mattson DJ, Reinhart DP, Schmidt WC, Holtmeier FK (1994). Bear use of whitebark pine seeds in North America. Proceedings of an international workshop on subalpine stone pines and their environment: the status of our knowledge.

[b52] Mattson DJ, Reinhart DP (1997). Excavation of red squirrel middens by grizzly bears in the whitebark pine zone. J. Appl. Ecol.

[b53] Mattson DJ, Blanchard BM, Knight RR (1991). Food habits of Yellowstone grizzly bears, 1977–1987. Can. J. Zool.

[b54] Mattson DJ, Blanchard BM, Knight RR (1992). Yellowstone grizzly bear mortality, human habituation, and whitebark pine seed crops. J. Wildl. Manag.

[b55] McNamara JM, Houston AI (1992). Risk-sensitive foraging: a review of the theory. Bull. Math. Biol.

[b56] Mealey SP, Pelton MR (1980). The natural food habits of grizzly bears in Yellowstone National Park, 1973–74. Bears: their Biology and Management: A Selection of Papers from the Third International Conference on Bear Research and Management, Moscow, USSR, June 1974.

[b57] Menéndez R, González Megías A, Hill JK, Braschler B, Willis SG, Collingham Y (2006). Species richness changes lag behind climate change. Proc. Biol. Sci.

[b58] Noyce KV, Garshelis DL (1997). Influence of natural food abundance on black bear harvest in Minnesota. J. Wildl. Manag.

[b59] Paralikidis NP, Papageorgiou NK, Kontsiotis VJ, Tsiompanoudis AC (2009). The dietary habits of the brown bear (*Ursus arctos*) in western Greece. Mamm. Biol.

[b60] Parmesan C (2006). Ecological and evolutionary responses to recent climate change. Annu. Rev. Ecol. Evol. Syst.

[b61] Patten DT (1963). Vegetational pattern in relation to environments in the Madison Range, Montana. Ecol. Monogr.

[b62] Pearse IA, Altermatt F (2013). Extinction cascades partially estimate herbivore losses in a complete Lepidoptera-plant food web. Ecology.

[b63] Pelton MR, Feldhamer GA, Thompson BC, Chapman JA (2003). Black bear. Wild mammals of North America: biology, management, and conservation.

[b64] Pinheiro J, Bates D, DebRoy S, Sarkar D (2013). http://cran.r-project.org/web/packages/nlme.

[b65] Podruzny S, van Manen FT, Haroldson MA, West K (2012). Use of diminished whitebark pine resources by adult female grizzly bears in the Taylor Fork area of the Gallatin National Forest, Montana, 2011. Yellowstone grizzly bear investigations: annual report of the Interagency Grizzly Bear Study Team, 2011.

[b66] Podruzny SR, Reinhart DP, Mattson DJ (1999). Fire, red squirrels, whitebark pine, and Yellowstone grizzly bears. Ursus.

[b67] Pyke GH, Pulliam HR, Charnov EL (1977). Optimal foraging: a selective review of theory and tests. Q. Rev. Biol.

[b68] R Development Core Team (2013). R: A language and environment for statistical computing.

[b69] Raffa KF, Aukema BH, Bentz BJ, Carroll AL, Hicke JA, Turner MG (2008). Cross-scale drivers of natural disturbances prone to anthropogenic amplification: the dynamics of bark beetle eruptions. Bioscience.

[b70] Rode KD, Robbins CT, Shipley LA (2001). Constraints on herbivory by grizzly bears. Oecologia.

[b71] Ryan CW, Pack JC, Igo WK, Billings A (2007). Influence of mast production on black bear non-hunting mortalities in West Virginia. Ursus.

[b72] Schrag AM, Bunn AG, Graumlich LJ (2008). Influence of bioclimatic variables on tree-line conifer distribution in the Greater Yellowstone Ecosystem: implications for species of conservation concern. J. Biogeogr.

[b73] Schwartz CC, Miller SD, Haroldson MA, Feldhamer GA, Thompson BC, Chapman JA (2003). Grizzly bear. Wild mammals of North America: biology, management, and conservation.

[b74] Schwartz CC, Haroldson MA, Cherry S (2006a). Reproductive performance of grizzly bears in the Greater Yellowstone Ecosystem, 1983–2002. Wildl. Monogr.

[b75] Schwartz CC, Haroldson MA, White GC (2006b). Survival of cub and yearling grizzly bears in the Greater Yellowstone Ecosystem. Wildl. Monogr.

[b76] Schwartz CC, Haroldson MA, White GC (2006c). Study area and methods for collecting and analyzing demographic data on the Yellowstone grizzly bear. Wildl. Monogr.

[b77] Schwartz CC, Haroldson MA, White GC (2010). Hazards affecting grizzly bear survival in the Greater Yellowstone Ecosystem. J. Wildl. Manag.

[b78] Schwartz CC, Fortin JK, Teisberg JE, Haroldson MA, Servheen C, Robbins C (2013a). Body and diet composition of sympatric black and grizzly bears in the Greater Yellowstone Ecosystem. J. Wildl. Manag.

[b79] Schwartz CC, Haroldson MA, Gunther KA, Robbins CT, White PJ, Garrott RA, Plumb GE (2013b). Omnivory and the terrestrial food web: Yellowstone grizzly diets. Yellowstone's Wildlife in Transition.

[b80] U.S. Fish and Wildlife Service (2007). Final conservation strategy for the grizzly bear in the Greater Yellowstone Area.

[b81] Van Daele LJ, Belant VG, Barnes JL (2012). Ecological flexibility of brown bears on Kodiak Island, Alaska. Ursus.

[b82] Van der Putten WH (2012). Climate change, aboveground-belowground interactions, and species' range shifts. Annu. Rev. Ecol. Evol. Syst.

[b83] Waddington JCB, Wright HE (1974). Late Quaternary vegetational changes on the east side of Yellowstone Park, Wyoming. Quatern. Res.

[b84] Warren MS, Hill JK, Thomas JA, Asher J, Fox R, Huntley B (2001). Rapid responses of British butterflies to opposing forces of climate and habitat change. Nature.

[b85] Warwell MC, Rehfeldt GE, Crookston NL (2007). Modeling contemporary climate profiles of whitebark pine (*Pinus albicaulis*) and predicting responses to global warming. Proceedings of the conference whitebark pine: a Pacific Coast Perspective 2006 August 27–31, Ashland, OR.

[b86] Welch CA, Keay J, Kendall KC, Robbins CT (1997). Constraints on frugivory by bears. Ecology.

[b87] Williams DW, Leibhold AM (2002). Climate change and the outbreak ranges of two North American bark beetles. Agric. For. Entomol.

