# Supplementary material for: Influence of whitebark pine decline on fall habitat use and movements of grizzly bears in the Greater Yellowstone Ecosystem
Source: Ecol Evol. 2014 Apr 22;4(10):2004–18. doi: 10.1002/ece3.1082 (PMC4063492; doi:10.1002/ece3.1082)
Supplement: Supplementary file 1 [file ece30004-2004-SD1.doc]

**Appendix S1**

**Assigning habitat to unsuccessful GPS fixes: rationale, methodology,**

**and evaluation of bias and accuracy**

**Rationale for assigning habitat for unsuccessful fixes**

Because of improved GPS technology, we observed an increasing trend in fix success over the study period. Annual success rates ranged from 62% to 94%, with a mean of 80% (Fig. S1). Information from the early part of the study period was vital to our investigation so we needed to include data from older transmitters, while accounting for this variation in fix success in our analyses. All of our habitat-related analyses involved only two habitat types (i.e., WBP versus non-WBP, or secure versus non-secure). Because of this simple dichotomy, we developed a method to assign habitat and weight for unsuccessful fixes: (1) to investigate if there was a bias in fix success for these habitat types; and (2) if no habitat bias was detected, to allow us to utilize unsuccessful fixes by assigning habitat and accounting for uncertainty in the assignment. By utilizing successful and unsuccessful fixes, each bear was equally represented by a full, albeit weighted, set of locations. For each bear, the weights were summed and divided by the total number of fix attempts to obtain an overall weight. These overall weights were used in regression analyses.

**Methodology and evaluation of habitat bias**

For all missing fixes, we calculated the midpoint between the previous and subsequent successful location. We constructed a buffer around this location with a diameter equal to the distance between the successful locations. We calculated the proportion of WBP and non-WBP habitats (and secure versus non-secure habitats) within the buffers. We then assigned each unsuccessful fix to the predominant habitat type within the buffer, but weighted that location by the corresponding proportion. For example, if the buffer was comprised of 85% WBP habitat and 15% non-WBP habitat, the location was assigned to WBP and weighted by 0.85. Successful fixes were weighted as 1. Among all unsuccessful fixes, 74% of buffers were comprised of only one habitat type for WBP versus non-WBP, and 81% were comprised of only one type for secure versus non-secure habitat. Therefore, these locations were also assigned a weight of 1.0.

Presumably, if either WBP or secure habitat imposed a bias in fix success, we would expect to observe a consistent pattern of under- or over-estimating use. When we compared proportional use of habitats based on successful fixes to proportional use based on unsuccessful-assigned fixes by bear, we observed high correlation for WBP habitat (*r* = 0.96, *P* < 0.001, *n* = 89) and secure habitat (*r* = 0.90, *P* < 0.001, *n* = 89), despite the fact that successful fixes far outnumbered unsuccessful fixes. The slopes of these relationships were very close to 1, indicating only a slight fix success bias against these habitats: 0.87 (95% CI: 0.84–0.91) for WBP habitat and 0.95 (95% CI: 0.93–0.97) for secure habitat.

**Evaluation of accuracy and effect on model results**

Using simulations, we tested the accuracy of the method by: (1) treating each successful fix as a missing fix, (2) applying the methodology to assign habitat type, and (3) comparing the assignment to the actual habitat type observed. Among 38,806 successful fixes (with previous and subsequent successful fixes available), assignment accuracy was 95%.

Finally, we evaluated whether these habitat assignments to unsuccessful fixes affected model results, based on simulations of the general linear model predicting the Manly-Chesson index for WBP habitat, with CONES + SEX + CONES × SEX + YEAR + CONES × YEAR as predictors. We ran 100 simulations whereby unsuccessful fixes were assigned values of 0 or 1 for WBP habitat at random (regardless of habitat within the buffer area) and compared the beta coefficients for these simulated models to those of our original model (which assigned habitat based on habitat within the buffer). Presumably, if unsuccessful fixes were numerous enough to affect model output, the simulated-random models would differ from the original model. In 100% of the simulations, all values for beta coefficients fell within the 95% CI of the beta coefficient from the original model. Overall, simulation analyses indicated our method for assigning habitat to unsuccessful fixes was highly accurate and unlikely to result in erroneous inference from modeling.

**Figure S1.1.** Mean fix success, by year, for GPS transmitters on grizzly bears monitored during 15 August–30 September, 2000–2011, Greater Yellowstone Ecosystem.
