# Supplementary material for: Influence of whitebark pine decline on fall habitat use and movements of grizzly bears in the Greater Yellowstone Ecosystem
Source: Ecol Evol. 2014 Apr 22;4(10):2004–18. doi: 10.1002/ece3.1082 (PMC4063492; doi:10.1002/ece3.1082)
Supplement: Supplementary file 2 [file ece30004-2004-SD2.doc]

Appendix S2. Coefficients for top-ranked models, based on Akaike Information Criterion (AIC_c_), predicting various grizzly bear responses, as a function of annual estimates of WBP cone production (CONES = median number of cones/tree on cone production transects), sex (0 = female, 1 = male), and year (0–11), Greater Yellowstone Ecosystem, 2000–2011. For linear models, response variables were mean Manly-Chesson standardized indices of selection for WBP habitat, for WBP habitat adjusted for mortality, and for secure habitat (any area ≥4.05 ha ≥500 m from an open or gated motorized road). For quantile regression models, response variable were median, 10^th^ percentile, and 90^th^ percentile of day-of-year for observations of WBP habitat use. For mixed-effects linear regression models, response variables were median daily movement distance and median activity radius.

|  |  |  |  |  |  |  | Random effect | |  | Likelihood ratio test^a^ | |
| --- | --- | --- | --- | --- | --- | --- | --- | --- | --- | --- | --- |
| Response | Parameter | β | SE | *t* | *P* |  | Intercept | Residual |  | Χ^2^ | *P* |
| Manly-Chesson index | Intercept | 0.734 | 0.059 | 12.42 | <0.001 |  | NA | NA |  | 0.83 | 0.36 |
| for WBP habitat | CONES | -0.002 | 0.005 | -0.32 | 0.75 |  |  |  |  |  |  |
|  | SEX | -0.181 | 0.081 | -2.22 | 0.03 |  |  |  |  |  |  |
|  | YEAR | -0.019 | 0.006 | -3.06 | 0.00 |  |  |  |  |  |  |
|  | CONES × SEX | 0.013 | 0.007 | 2.02 | 0.05 |  |  |  |  |  |  |
|  |  |  |  |  |  |  |  |  |  |  |  |
| Manly-Chesson index | Intercept | 0.734 | 0.059 | 12.49 | <0.001 |  | NA | NA |  | 0.60 | 0.44 |
| for impact-adjusted WBP habitat | CONES | -0.002 | 0.005 | -0.36 | 0.72 |  |  |  |  |  |  |
|  | SEX | -0.189 | 0.081 | -2.33 | 0.02 |  |  |  |  |  |  |
|  | YEAR | -0.017 | 0.006 | -2.68 | 0.01 |  |  |  |  |  |  |
|  | CONES × SEX | 0.014 | 0.007 | 2.09 | 0.04 |  |  |  |  |  |  |
|  |  |  |  |  |  |  |  |  |  |  |  |
| Day-of-year | Intercept | 249.000 | 1.240 | 200.73 | <0.001 |  | NA | NA |  | NA | NA |
| (median) | CONES | 0.019 | 0.125 | 0.15 | 0.88 |  |  |  |  |  |  |
|  | SEX | -3.000 | 1.788 | -1.68 | 0.09 |  |  |  |  |  |  |
|  | YEAR | 0.778 | 0.201 | 3.87 | <0.001 |  |  |  |  |  |  |
|  | CONES × SEX | 0.300 | 0.139 | 2.15 | 0.03 |  |  |  |  |  |  |
|  | CONES × YEAR | -0.030 | 0.018 | -1.65 | 0.10 |  |  |  |  |  |  |

^a^ Likelihood ratio test between nested fixed-effects and mixed-effects models to determine when random effects were needed to improve model fit.

|  |  |  |  |  |  |  | Random effect | |  | Likelihood ratio test^a^ | |
| --- | --- | --- | --- | --- | --- | --- | --- | --- | --- | --- | --- |
| Response | Parameter | β | SE | *t* | *P* |  | Intercept | Residual |  | Χ^2^ | *P* |
| Day-of-year | Intercept | 232.000 | 1.063 | 218.21 | <0.001 |  | NA | NA |  | NA | NA |
| (10th percentile) | CONES | -0.025 | 0.088 | -0.29 | 0.77 |  |  |  |  |  |  |
|  | SEX | -2.000 | 1.217 | -1.64 | 0.10 |  |  |  |  |  |  |
|  | YEAR | 0.330 | 0.104 | 3.19 | <0.001 |  |  |  |  |  |  |
|  | CONES × SEX | 0.077 | 0.102 | 0.75 | 0.45 |  |  |  |  |  |  |
|  |  |  |  |  |  |  |  |  |  |  |  |
| Day-of-year | Intercept | 269.000 | 0.703 | 382.82 | <0.001 |  | NA | NA |  | NA | NA |
| (90th percentile) | CONES | -0.087 | 0.065 | -1.34 | 0.18 |  |  |  |  |  |  |
|  | SEX | -2.000 | 1.487 | -1.35 | 0.18 |  |  |  |  |  |  |
|  | YEAR | 0.136 | 0.091 | 1.50 | 0.13 |  |  |  |  |  |  |
|  | CONES × SEX | 0.176 | 0.103 | 1.71 | 0.09 |  |  |  |  |  |  |
|  |  |  |  |  |  |  |  |  |  |  |  |
| Log (daily movement distance) | Intercept | 0.196 | 0.144 | 1.36 | 0.17 |  | 0.46 | 1.65 |  | 115.30 | <0.001 |
|  | CONES | 0.007 | 0.013 | 0.56 | 0.58 |  |  |  |  |  |  |
|  | SEX | 0.044 | 0.229 | 0.19 | 0.85 |  |  |  |  |  |  |
|  | CONES × SEX | 0.002 | 0.019 | 0.10 | 0.92 |  |  |  |  |  |  |
|  |  |  |  |  |  |  |  |  |  |  |  |
| Log (activity radius) | Intercept | 1.148 | 0.174 | 6.60 | <0.001 |  | 0.53 | 0.88 |  | 699.40 | <0.001 |
|  | CONES | -0.011 | 0.014 | -0.80 | 0.43 |  |  |  |  |  |  |
|  | SEX | 0.247 | 0.241 | 1.02 | 0.31 |  |  |  |  |  |  |
|  | YEAR | 0.004 | 0.019 | 0.20 | 0.84 |  |  |  |  |  |  |
|  | CONES × SEX | 0.032 | 0.020 | 1.62 | 0.11 |  |  |  |  |  |  |
|  |  |  |  |  |  |  |  |  |  |  |  |
| Manly-Chesson index | Intercept | 0.590 | 0.067 | 8.83 | <0.001 |  | NA | NA |  | 0.12 | 0.73 |
| for secure habitat | CONES | 0.012 | 0.008 | 1.55 | 0.13 |  |  |  |  |  |  |
|  | SEX | 0.014 | 0.083 | 0.17 | 0.87 |  |  |  |  |  |  |
|  | YEAR | 0.003 | 0.010 | 0.29 | 0.78 |  |  |  |  |  |  |
|  | CONES × SEX | 0.000 | 0.009 | -0.01 | 0.99 |  |  |  |  |  |  |
|  | CONES × YEAR | -0.002 | 0.001 | -1.97 | 0.06 |  |  |  |  |  |  |

^a^ Likelihood ratio test between nested fixed-effects and mixed-effects models to determine when random effects were needed to improve model fit.
